# Rethinking students’ mental health assessment through GHQ-12: evidence from the IRT approach

**DOI:** 10.1186/s40359-024-01808-4

**Published:** 2024-05-29

**Authors:** Anna Comotti, Teresa Barnini, Alice Fattori, Maria Emilia Paladino, Michele Augusto Riva, Matteo Bonzini, Michael Belingheri

**Affiliations:** 1 Occupational Medicine Unit, Fondazione IRCCS Ca’ Granda Ospedale Maggiore Policlinico, Milan, Italy; 2 Unit of Occupational Health, Fondazione IRCCS San Gerardo dei Tintori, Monza, Italy; 3 School of Medicine and Surgery, University of Milano-Bicocca, Monza, Italy; 4 Department of Clinical Science and Community Health, University of Milan, Milan, Italy

**Keywords:** Item response theory, General Health Questionnaire, University students, Covid-19, Occupational health surveillance

## Abstract

**Background:**

The General Health Questionnaire-12 (GHQ-12) is a widely used screening tool for mental health assessment however its traditional scoring methods and cutoffs may not adequately capture the mental health complexities of younger populations.

**Methods:**

This study explores GHQ-12 responses from a sample of university students. Possible differences in means scores considering gender, age, academic field and degree course were assessed through t-test or one-way ANOVA as appropriate. To deeper understanding different levels of severity and individual item impact on general distress measurement, we applied Item-Response-Theory (IRT) techniques (two-parameters logistic model). We compared students’ population with a population of workers who underwent a similar psychological evaluation.

**Results:**

A total of 3834 university students participated in the study. Results showed that a significant proportion (79%) of students reported psychological distress. Females and younger students obtained significantly higher average scores compared to others. IRT analysis found item-specific variations in mental distress levels, with more indicative items for short-term fluctuations and potential severe mental health concerns. Latent class analysis identified three distinct subgroups among students (including 20%, 37%, 43% of the participants respectively) with different levels of psychological distress severity. Comparison with a population of adults showed that students reported significantly higher scores with differences in the scale behavior.

**Conclusion:**

Our results highlighted the unique mental health challenges faced by students, suggesting a reevaluation of GHQ-12 applicability and cutoff scores for younger populations, emphasizing the need for accurate instruments in mental health evaluation.

**Supplementary Information:**

The online version contains supplementary material available at 10.1186/s40359-024-01808-4.

## Background

The 12 item General Health Questionnaire (GHQ-12; [1]) is a reliable and valid screening tool for detecting psychological impairment as well as short-term changes in mental health [2, 3]. Due to its easy administration and brevity, GHQ-12 has been translated in several languages and equally adopted in different settings, countries, and populations [4, 5].

GHQ-12 can be scored by adopting the binary scale (0-0-1-1) or the 4-point Likert-type scale (0-1-2-3) methods and responses to all items are summed up to a total score ranging from 0 to 12 (binary scale) or to 0 to 36 (Likert scale), with higher scores indicating more impairment [6]. A score above a certain cut-off (typically 3/4 and 13/14 for bimodal and Likert scale respectively) usually indicates the presence of psychological distress [1, 7, 8].

Goldberg and colleagues already noted that the cut off scores may vary among different populations and that indiscriminately using the same scoring method could lead to erroneous classification of mental illness’ severity [9]. Each item may in fact express different level of the psychological distress yet such potential difference in items contribution may be lost by counting each item the same within the overall score.

Item Response Theory (IRT) provides more details about individual items and could be considered a more suitable tool than the usual methodologies based on Classical Test Theory (CTT). While the CTT consider a single overall score, where each item counts the same and the complexity of underlying traits is lost, the strength of IRT technique lies in its focus on items rather than individual scores.

Nowadays, while only few studies apply IRT technique to interpret GHQ-12 scores, the vast majority still use the total score and relative cut-offs to assess mental health status in various settings. For instance, examples of mental health evaluation through GHQ-12 are available for adults [10], adolescents [11], clinical populations [12], university students [13, 14], healthcare workers [15]. Moreover, GHQ-12 was frequently used as a tool for the medical surveillance to assess the mental health status and to detect psychological impairment among workers exposed to stressors during their work activities. For example, it has been extensively adopted during the Covid-19 pandemic among specific samples particularly affected such as health-care workers [8, 16, 17] and younger adults [18, 19].

Aim of this study was to analyze data from a sample of university students through the IRT approach, since to our knowledge the GHQ-12 in this specific population has always been assessed using traditional score methods and no studies adopted the IRT approach to assess GHQ-12 within young adults. We wanted to investigate how the mental health status was captured by GHQ-12 among our population of young adults, identifying different levels of severity and quantifying the impact of each item on the measurement of general distress. Furthermore, we compared students’ data with a sample of healthcare workers (HCWs) whose mental health was assessed in the same period, in the same city, by the same questionnaire and previously analyzed through latent class IRT models [20]. Such results already highlighted the potential of IRT in determining different levels of severity with interesting clinical application which may lead to a more efficient usage of GHQ-12.

## Methods

The present study involved students of a big-sized Northern Italian university. Data were collected from April 2021 to May 2021 using a self-administered online survey. All the students, without any exclusion criteria, received the online link through institutional email and filled the questionnaire at their home; participation was on a voluntary basis. The study was approved by the Independent Ethics Committee of the University of Milano-Bicocca (n. 580/2021 of February 16, 2021).

The survey first investigated a wide range of variables including students’ socio-demographic characteristics, academic field (i.e., economic and law, sanitary, scientific, humanistic) and degree courses (i.e., bachelor programs, master programs, single-cycle programmes, doctoral research, professional master programmes and specialization schools).

Psychological distress was measured using the Italian validated version of the General Health Questionnaire-12 [21], using the standard dichotomous method, with a clinical cut off score set at 4.

Descriptive statistics were reported using frequencies and percentage for categorical data and mean and standard deviation or median and IQR for continuous variables.

Student t-test or one-way ANOVA as appropriate investigated possible differences in GHQ-12 mean scores among sub-groups (gender, degree course and academic field).

Internal consistency was assessed by Cronbach’s alpha coefficient.

We used IRT techniques to analyze the items responses. We applied the two parameters (2-PL) logistic model for modelling the probability of giving answer equal to 1, which corresponds to the response categories “less than/same as usual” or “more/much more than usual” for positively and for negatively phrased items respectively, depending on the level of latent trait $\vartheta$ (psychological distress) and items’ threshold (or difficulty) and discriminating parameters. Item Characteristics Curves (ICCs) resulted from 2-PL model were graphically represented. To detect different level of mental health distress severity, we performed a latent class analysis (LCA) through the LC-IRT model for dichotomous responses, where every level of $\vartheta$ (assumed to have discrete distribution) corresponds to a latent class of subjects in the population. We used the 2-PL model in its discrete version. The number of latent classes was chosen according to fit indices such BIC. For each class, we calculated the percentage of answers equal to 1 to each item and the average GHQ-12 score.

Details of 2-PL LC-IRT models are described in the Supplementary Material.

To better identify the characteristics of the scale within a population of students, we compared GHQ-12 scores and IRT parameters estimation with results from the same questionnaire administered in a population of healthcare workers whose psychological distress was measures thorough GHQ-12 in the same city, the same period and during a similar health surveillance program. Furthermore, considering the pandemic context in which the survey was conducted, healthcare workers were not involved in the lockdown and therefore the comparison between these two populations provided an interesting contrast in terms of mental health. We tested differences in mean through t-test, differences in frequencies of scorings above cut-off through Chi-square test and we graphically represented the ICCs of the same 2-PL model performed on adults’ population.

Data were analyzed using the R software [22], and a *p*-value < 0.05 was considered as statistically significant.

## Results

A total of 3834 students participated in the survey. Participants were predominately female (*N* = 2837, 74%), with median age of 22 (IQR = 20–24), attending human faculties (*N* = 1450, 38%), enrolled in bachelor’s degree courses (*N* = 2115, 55%) with similar distribution compared to the eligible student population (i.e., around 33,000; [23]). Demographic characteristics of the study population are shown in Table 1.

**Table 1:** Descriptive statistics. Frequencies and percentage by gender, academic field and degree course. GHQ-12 results (mean, sd) in the total sample and by subgroups. (t-test or one-way ANOVA *p < .05, **p < .01, ***p < .001)

	*N* (%)	GHQ-12 mean score (sd)
**TOTAL SAMPLE**	3834	7.2 (3.8)
**Gender** ***		
Male Female	997 (26) 2837 (74)	6.3 (3.9) 7.6 (3.7)
Academic field*		
Economics and Law Medicine and Surgery Sciences Human Sciences	1068 (28) 323 (8) 993 (26) 1450 (38)	7.5 (3.8) 7.0 (3.9) 7.0 (3.9) 7.3 (3.7)
**Degree course** ***		
Bachelor programmes Master programmes Single-cycle programmes Doctoral research (PhD) Specialization Schools Professional Master programmes	2115 (55) 1019 (27) 495 (13) 130 (3) 37 (1) 38 (1)	7.5 (3.7) 6.9 (3.8) 7.4 (3.8) 6.4 (4.0) 4.8 (4.1) 4.9 (3.5)

Regarding dichotomous GHQ-12, respondents reported an average total score equal to 7.2 (sd = 3.8) and a percentage of 79% scorings above relevant cut-off (equal to 4). Females showed significantly higher psychological distress than males with means equal to 7.6 (sd = 3.7) and 6.3 (sd = 3.9) respectively. Statistically significant differences occurred among areas of study (with lower scores among Medicine and Surgery and Science students) and degree courses in particular students enrolled in the first years (bachelor programmes) obtained significantly higher scores (mean = 7.5, sd = 3.7) compared to others in subsequent years (Table 1).

GHQ-12 answers reported good internal consistency with Cronbach alpha equal to 0.88 with 95%CI=(0.88, 0.89).

**Fig. 1:**
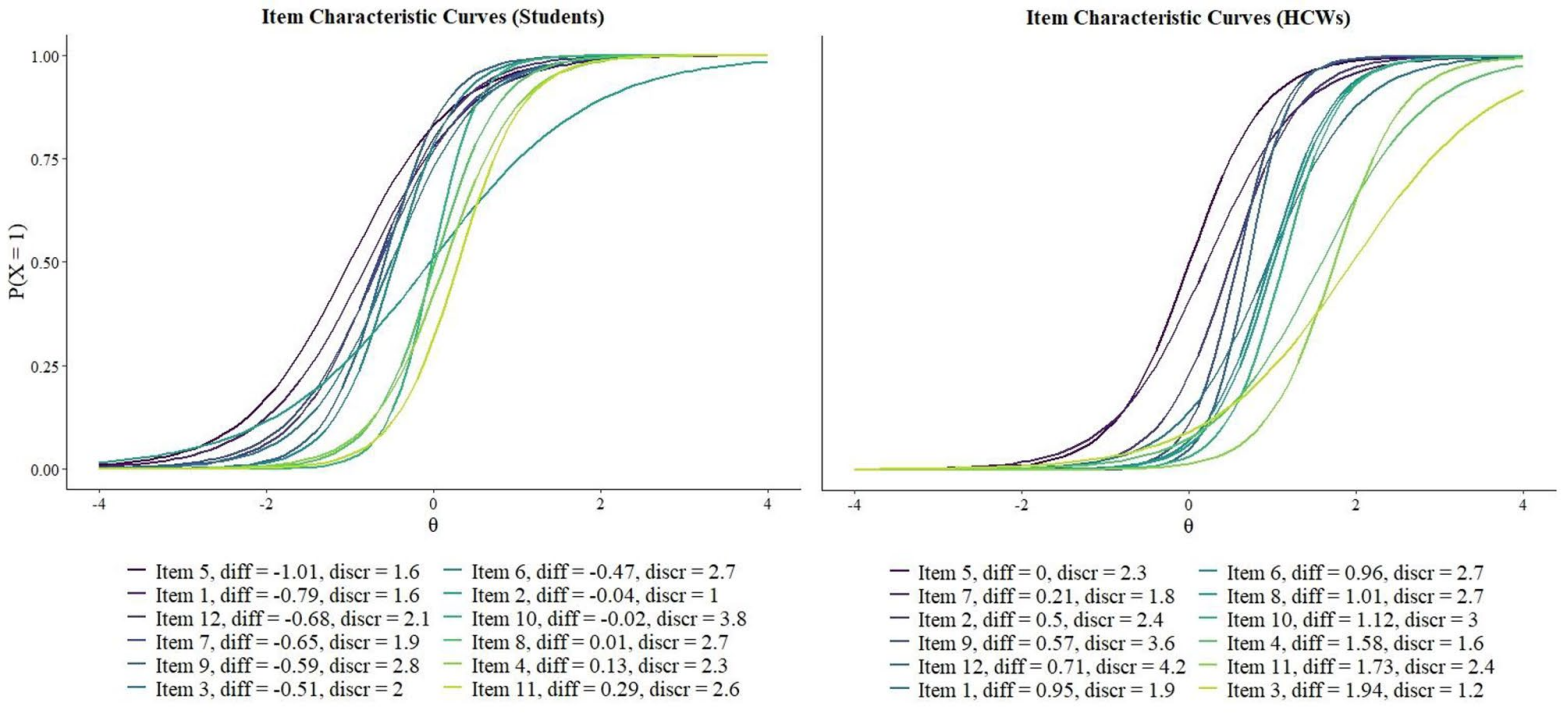
ICCs in the two populations: students (left) and HCWs (right). Threshold and discrimination parameters are reported below the curves

IRT parameters showed in Fig. 1 indicated many negative threshold parameters, meaning that the level of distress needed to give answer 0 or 1 with the same probability was low. Item 5 (feeling constantly under strain) and Item 11 (thinking of self as worthless) showed the lowest and highest threshold respectively. Item 10 (losing confidence) with the highest discriminating parameter was the best in discriminating students with different levels of distress, while the item regarding sleep habits (Item 2) discriminated less than the others (its ICC was the flattest in Fig. 1).

The LCA reached the lowest values of BIC with *k* = 3 latent classes (BIC equal to 47,620, 45,623, 45,842 for k = 2,3,4 respectively), i.e., it classified students in three groups with increasing level of psychological distress, whose distribution is shown in Table 2 together with the percentages of 1-answers to the items in each class. A high level of distress ($\vartheta$=2.6) was assigned to the 43% of the subjects and the 37% expressed lower level of distress ($\vartheta$=0.8) with the most frequent (>50%) 1-score answers for Item 5 (feeling constantly under strain), Item 1 (able to concentrate), Item 9 (feeling unhappy and depressed) Item 12 (feeling reasonably happy), which were indeed the lowest threshold items. A smaller (20%) group of students expressed no sign of impairment ($\vartheta$=-0.1). GHQ-12 average scores calculated for each class increased as ϑ level increased.

**Table 2:** LCA results. Estimated level of ϑ, a-posteriori percentage of subjects assigned to each class, corresponding GHQ-12 mean (sd) and percentage of 1-answers given to each item

	Class 1	Cass 2	Class 3
ϑ** level**	-1.0	0.8	2.6
**% of subjects**	20%	37%	43%
**GHQ-12 mean (sd)**	1.5 (1.1)	6.2 (1.6)	10.8 (1.1)
	**% 1-answers**		
**Item 1**	26	69	93
**Item 2**	18	44	73
**Item 3**	15	60	93
**Item 4**	3	28	82
**Item 5**	34	75	95
**Item 6**	7	62	97
**Item 7**	19	66	94
**Item 8**	3	31	88
**Item 9**	10	69	97
**Item 10**	1	29	94
**Item 11**	1	18	78
**Item 12**	18	69	95

When compared to a population of adults (990 HCWs), whose psychological distress was measured with the same questionnaire, during the same period and in the same city, students’ scorings and percentage above relevant cut-off were significantly higher (means 7.2 vs 3.2 and percentage 79% vs 37% for students and HCWs respectively, Table S1). IRT analyses performed on both populations showed differences in the estimation parameters (Fig. 1). The ICCs of students’ data were much left-shifted with respects to HCWs’, but the threshold parameters were similarly ordered, except for Item 3 (feeling useful). Students’ and HCWs’ levels of distress were discriminating mostly by Item 10 (losing confidence) and Item 12 (feeling reasonably happy) respectively and for both population by Item 9 (feeling unhappy and depressed).

## Discussion

To the best of our knowledge, this is the first study to assess university students’ mental health through GHQ-12 and IRT approach. In line with previous research [18, 19, 24–29], the present study confirms that the prevalence of psychological distress among Italian university students is widespread. In our sample of 3834 students, we found that almost 79% had a GHQ-12 score above cut off, with higher average scores in younger students. These tendency was already found among both Italian [10, 18, 26, 28] and worldwide studies [30, 31], where younger subjects showed higher scores than older populations, indicating higher prevalence of distress and common mental disorders.

Adolescence and young adulthood are one of the most common onset periods for major psychiatric disorders [32–34]. These are crucial times of biological and social transition, characterized by changes, pressures and choices about career and intimate relationship [24]. In this phase of development, people prepare for their adult life and make crucial decisions that will define who they are [35]. For many young people, this delicate phase coincides with the years of university studies. School and academic life implies opportunities and risks and are normally considered as an important source of tension: indeed, leaving home, dealing with new social and educational contexts, first financial difficulties, academic pressures and so on could render the university years as a stressful time for young women and men [32]. In this scenario/framework, it is important to consider that the GHQ-12 assessed the respondent’s current state and asks if that differs from the usual state. It is therefore more sensitive to short-term mental changes and psychiatric disorders but not to the long-standing attributes of the respondents. In this sense, it can be considered more as a measure of state (e.g. a relative temporary condition reactive to events) than a measure of trait (e.g. a personality aspect, reasonably stable over long periods of time) [30]. These observations could provide a possible explanation for such higher and above cut-off scores in young adults’ population: the younger you are, the more you go through a period of changes and stresses, the more the GHQ-12 is sensitive to these short-term mental changes.

Our sample showed higher scores in females than in males, underlying greater psychological distress and pressure. These findings are consistent with previous research which pointed out that female usually show higher levels of both academic and clinical stress than male [36], with a greater number of symptoms of psychological distress and depression [37]. While some of this gender differences may be accounted genetically, with hormonal changes and fluctuations working as a trigger for depression [38], a possible alternative explanation for these findings argues that men are usually less likely to talk about their feelings or seek for help for mood problems [39]. Therefore, the greater prevalence of psychological distress among women could be due to the fact that women are more able to express depressive and distress symptoms more easily [40].

In our study, first-year students reported higher stress than later years students. This result is in line with literature [27] suggesting that practice and social experiences promote students’ adaptability and enhance their strategies to cope with stress and other difficulties during university years [41].

Furthermore, even though small, significant differences in GHQ-12 scores occurred considering the academic field of study. Students of medicine and sciences obtained significantly lower scores compared to other degree courses reflecting a slightly better psychological status while students of economics and law reported worse mental health. While the literature on the mental health of medical students, especially assessed during the pandemic, is extensive, there is a lack of comparative analysis with other degree programs [42]. Therefore, further studies investigating differences in the students’ mental health considering the type of degree course would be necessary.

As we have seen, the GHQ-12 scale administered to young people may be excessively sensitive to the many changes and mood fluctuations typical of age. We proposed to analyze GHQ-12 data through the Item Response Theory (IRT) statistical model, as it provides more details about individual items and could be considered a more suitable tool than the usual methodologies based on CTT. The use of IRT techniques and the focus on the items’ characteristics allowed us to deeply investigate how the mental health status was captured by GHQ-12 in our specific population of young adults, identifying different levels of severity (given by the item difficulty) and quantifying the impact of each item on the measurement of general distress.

First, the IRT analysis allowed to highlight that, among young adults, the level of psychological distress needed to give with the same probability positive or negative answers was generally low, thus showing a high tendency to report psychological difficulties.

In particular, Item 5 (feeling constantly under strain) could be considered as the “easiest” item (i.e. lowest threshold), indicating that the higher prevalence of our sample easily reported critical changes in perceived stress levels during everyday life. University years are generally recognized as a high stress-period, characterized by several stress factors (e.g., academic, financial, and social stress factors) impacting on continuous fluctuations and changes in stress levels [43]. Moreover, the specific data collection period during Covid-19 pandemic and restrictions may have contributed to increase perceived stress levels. Indeed, young adults experienced additional stress due to sense of loneliness and future uncertainty, since profound changes occurred in their social and everyday habits [44].

Feelings of self-worthlessness and loss of self-confidence frequently characterized major psychological disorders, such as depression and anxiety. Our IRT results reflected these clinical observations: first, the distress level needed to give a positive or negative answer to item 11 (thinking of self as worthless) with the same probability was high, emphasizing that this critical psychological alteration could thus be considered due to a severe psychological disorder more than to frequent and continuous short-term mental changes. Moreover, the loss of self-confidence (Item 10) resulted to best discriminate students with different levels of distress, pointing out how having a high self-esteem and confidence in one’s own abilities helps in dealing with difficulties and in facing stressors.

Interestingly, students’ sleep habits (Item 2) showed an anomalous behavior; indeed Item 2 resulted to be the worst discriminating and the least informative item about the students’ wellbeing and this result is partly in contrast with literature that usually highlight the strong impact of stress on sleep quality. However, some studies highlighted that university students usually have a higher prevalence of sleep disturbances [45, 46], probably due to the stressful period they go through. It is thus possible that this specific population have a general poor sleep quality and consequently haven’t experienced great changes or alterations during the last period.

The IRT analysis also allowed the classification of our sample according to different psychological impairment levels: the first class includes those subjects without distress (i.e. with almost all responses equal to 0); the second class includes subjects with low severity psychological distress as the percentages of responses equal to 1 where high only for almost half of the item; the third class includes those young adults with psychological impairment.

Interestingly, students’ prevalence classified in the third class is about 43%, showing that the higher prevalence of our sample pertains to the first two classes. This result contributes to the reading of the GHQ-12 as a measure of state, where most of the psychological alterations are due to short-term mental changes (class 2), frequently occurring during the young adulthood, rather than to stable psychological disorders (class 3).

The two gravity classes (i.e., second and third class) differ in the item response pattern with regards to the capability in decision making (Item 4), facing problems (Item 8), and experiencing feeling worthless (Item 11), with class 3 subjects reporting a higher prevalence of impairment and class 2 showing an opposite answers distribution. Interestingly, these are all psychological difficulties frequently associated with major psychological disorders, such as depression, anxiety, and stress, highlighting the difference in severity and impact of the psychological alteration between the two classes.

Taken together, all these observations lead us to reflect about the use of the GHQ-12 scale with younger population. Although the GHQ-12 is a well-established clinical tool to screen and to assess the greatest psychological difficulties occurred in the latest period, using the same cut-off scores for younger and older population would seem to make the test excessively sensitive to the many moods’ alteration/fluctuation typical of transitional ages, which thus risk to be classified as clinically significant. The IRT analysis could overcome these problems, providing different weight to each item, and expressing different severity of the psychological impairment measured by the test.

A brief comparison between university students and an adult healthcare workers population confirmed the presence of higher stress levels among younger adults. Indeed, the percentage of HCW above GHQ-12 cut-off resulted much lower than that of university students (36% vs. 79% respectively) and, in addition, the IRT analysis, confirmed that students tended to report a higher severity of the symptoms associated with common mental health (i.e., each item has lower threshold parameters). Threshold parameters indicated that questions about feelings of worthless (Item 11), capacity of make decisions (Item 4) and loss of confidence (item 8) most affected the psychological wellbeing in both populations. Interestingly, feelings of useless (Item 3) showed the greatest difference in threshold parameters and significant difference in discrimination parameter between the two groups. This is probably due to the fact that, especially in the period when the questionnaire was administered, students and health care workers had significantly different roles: healthcare workers continued their work in the hospital, playing an important social role, while students remained at home because of the restrictions. A loss of confidence in their usefulness could have significantly affected their mental health in different ways. The differences observed between the two populations confirmed what Goldberg already argued regarding the use of the same scoring method in diverse populations, which might lead to an incorrect classification of severity [9]. The validity of the GHQ-12 scale has widely been demonstrated in adults, and the cutoffs have been established based on adult populations. From our comparison, it emerges that further validation studies in younger populations would be necessary for a more appropriate use of this questionnaire.

Our study has some limitations. First, despite the large sample size, participation rate was low and this may entail a self-selection bias. However, our sample is representative of the general university population [23] with heterogeneous sub-groups considering gender, degree courses and academic fields. Second, the data in this study were collected through self-administered online surveys relying on participants’ self-reporting, which can introduce response bias [47] as participants may have under or over reported their mental health status due to social desirability bias. Lastly, data collection occurred during the Covid-19 pandemic, which could have had a significant impact on participants’ mental health. Nevertheless, our results agree with the present literature on mental health among young people, measured before and during Covid-19 pandemic. Moreover, the present study allowed a deeper investigation of the GHQ-12 using IRT methods, suggesting potential adjustments in cutoff scores for younger populations, which can impact mental health assessment practices. To our knowledge, this is the first time that this technique is applied on GHQ-12 among university students.

## Conclusion

The IRT approach could have a significant impact in other different settings in which the GHQ-12 is commonly used, providing a more suitable tool than the usual methodologies. Indeed, the GHQ-12 scale is frequently used in medical surveillance to assess and monitor the mental health status and the psychological impairment of workers in different occupational settings. Since the use of the same cut-off scores with the traditional CCT approach for younger and older population proved to be inefficient, the IRT could be useful to better assess the mental health status in different populations of workers, providing more suitable information about the psychological impairment that could be important for subsequent preventive and rehabilitation measures. Future research could further investigate differences in the GHQ-12 scores between younger and older populations, to identify a more suitable cut-off for younger people.

### Electronic supplementary material

Below is the link to the electronic supplementary material.

Supplementary Material 1

## Data Availability

Data are available upon reasonable request from the corresponding author AC.

## Abbreviations

2–PL: Two parameters Logistic
BIC: Bayesian Information Criterion
CTT: Classical Test Theory
GHQ-12: 12 items General Health Questionnaire
ICC: Item Characteristic Curve
IRT: Item Response Theory
LC: Latent Class
LCA: Latent Class Analysis
