# Supplementary material for: Rethinking students’ mental health assessment through GHQ-12: evidence from the IRT approach
Source: BMC Psychol. 2024 May 29;12:308. doi: 10.1186/s40359-024-01808-4 (PMC11134724; doi:10.1186/s40359-024-01808-4)
Supplement: Supplementary file 1 — Supplementary Material 1 [file 40359_2024_1808_MOESM1_ESM.docx]

**TITLE**

**Rethinking students’ mental health assessment through GHQ-12: evidence from the IRT approach**

**SUPPLEMENTARY MATERIAL**

We specify the IRT models.

2-PL model

We implemented the two parameters logistic model, which expresses the probability that an individual *i* at a given level of the latent trait $\vartheta_{i}$will give an answer equal to 1 to item *j* as

$P(Y_{ij}=1\left| \theta_{i} \right)=\frac{e^{\lambda_{j}(\theta_{i}-\beta_{j})}}{1+e^{\lambda_{j}(\theta_{i}-\beta_{j})}}$,

where $\beta$_j_ and $\lambda$_j_ are the threshold (or difficulty) and the discriminating parameters of the item *j* respectively.

In detail,

- $\vartheta_{i}$ is the value of the latent variable $\Theta$ observed for individual *i*. It indicates the point on the latent continuum where individual *i* is located; it is measured in logit.
- $\beta$_j_is the item difficulty or threshold. If $\vartheta$_i_$= \beta$_j_ then $P\left( Y_{ij}=1 \right)=0.5,$ if $\vartheta$_i_$> \beta$_j_ then $P\left( Y_{ij}=1 \right)>0.5$ and if $\vartheta$_i_$< \beta$_j_ then $P\left( Y_{ij}=1 \right)<0.5.$ The item difficulty represents the level of latent trait for which one has the 50% probability of responding “correctly” (=1) to that item.
- $\lambda$_j_ is the discrimination parameters. It estimates the capacity of that item to distinguish between subjects with different latent trait level.

LC-IRT model, (Bartolucci, 2007)

For the latent class analysis, we applied the 2-PL model in its discrete version. The main assumption is that the population under study is composed of homogeneous classes (or sub-populations) of individuals who have very similar unobservable characteristic. In this case, the IRT model is expressed as a latent class model under the discreteness assumption for $\Theta_{i}$, i.e., every random variable $\theta_{i},$ *i=1,…n* is assumed to have a discrete distribution with support points $\xi_{1},\ldots, \xi_{n}$ and corresponding weights $\pi_{1},\ldots,\pi_{k}$. Each $\pi_{v}$ represents the probability that a subject belongs to class *v*, $\pi_{v}=P\left( \Theta_{i}=\xi_{v} \right),$ where $\sum_{v} \pi_{v}=1; \pi_{v}\geq0.$

The number of latent class *k* is assumed a priori, on the basis of theoretical knowledge or substantial reason, or selected by comparing the fit of the model under different values of *k.*

REFERENCE

Bartolucci, F. (2007). A class of multidimensional IRT models for testing unidimensionality and clustering items. *Psychometrika*, 72, 141-157.

**Table S1. Comparing two populations: GHQ-12 average scores and percentage above relevant cutoff in students and HCWs. ***p<.001 (t-test or Chi-square test)**

|  | **Students**  **N=3834** | **HCWs**  **N=990** |
| --- | --- | --- |
| **GHQ-12 Mean (sd)***** | 7.2 (3.8) | 3.1 (3.3) |
| **GHQ-12 Median (IQR)** | 8 (4-11) | 2 (0-5) |
| **scores > cutoff***** | 79% | 37% |
